# Single-crown restorations supported by short implants (6 mm) compared with standard-length implants (13 mm) in conjunction with maxillary sinus floor augmentation: a randomized, controlled clinical trial

**DOI:** 10.1186/s40729-021-00348-5

**Published:** 2021-07-16

**Authors:** Helle Baungaard Nielsen, Søren Schou, Niels Henrik Bruun, Thomas Starch-Jensen

**Affiliations:** 1 Department of Oral and Maxillofacial Surgery, Aalborg University Hospital, Aalborg, Denmark; 2 Department of Periodontology, School of Dentistry, University of Copenhagen, Copenhagen, Denmark; 3 Unit of Epidemiology and Biostatistics, Aalborg University Hospital, Aalborg, Denmark

**Keywords:** Bone augmentation, Bone substitutes, Dental implants, Dental prosthesis, Randomized, Controlled, Clinical trial

## Abstract

**Background:**

The purpose of the present study was to test the H0-hypothesis of no difference in the clinical and radiographical treatment outcome of single-crown restorations supported by short implants compared with standard length implants in conjunction with maxillary sinus floor augmentation (MSFA) after 1 year of functional implant loading. Forty patients with partial edentulism in the posterior part of the maxilla were randomly allocated to treatment involving single-crown restorations supported by short implants or standard length implants in conjunction with MSFA. Clinical and radiographical evaluation were used to assess survival of suprastructures and implants, peri-implant marginal bone loss (PIMBL), biological, and mechanical complications.

**Results:**

Both treatment modalities were characterized by 100% survival of suprastructures and implants after 1 year. Mean PIMBL was 0.60 mm with short implants compared with 0.51 mm with standard length implants after 1 year of functional loading. There were no statistically significant differences in survival of suprastructure and implants, PIMBL, and mechanical complications between the two treatment modalities. However, a higher incidence of biological complications was associated with standard length implants in conjunction with MSFA.

**Conclusion:**

Within the limitations of the present study, it can be concluded that single-crown restorations supported by short implants seems to be comparable with standard length implants in conjunction with MSFA. However, long-term studies are needed before final conclusions can be provided about the two treatment modalities.

**Trial registration:**

Clinicaltrials.Gov ID: NCT04518020. Date of registration: August 14, 2020, retrospectively registered.

## Background

Prosthetic rehabilitation of the partially edentulous posterior part of the maxilla with an implant-supported fixed prosthesis is frequently compromised or impossible due to atrophy of the alveolar process or pneumatization of the maxillary sinus following tooth loss. Thus, vertical alveolar ridge augmentation is often necessary before or in conjunction with placement of implants. Maxillary sinus floor augmentation (MSFA) applying the lateral window technique is the most commonly applied surgical procedure to increase the vertical alveolar ridge height of the posterior part of the maxilla, and high survival rate of suprastructure and implants have been reported in several systematic reviews and meta-analyses [1–5]. Autogenous bone graft alone or in combination with a bone substitute is frequently used as grafting material [1, 2, 6]. However, harvesting of autogenous bone grafts is associated with a supplementary surgical procedure, risk of donor site morbidity, and unpredictable resorption of the grafting material [2, 6–10].

Placement of short implants has therefore been advocated as an alternative treatment modality for prosthetic rehabilitation of the partially edentulous posterior part of the maxilla to simplify the surgical procedure and eliminate the need for bone harvesting [11]. A newly published systematic review and meta-analysis demonstrated no statistically significant differences in implant survival or peri-implant marginal bone loss (PIMBL) after placement of short implants (≤ 8 mm) compared with standard length implants (> 8 mm) in conjunction with MSFA after 3 years of functional implant loading [12]. These results are in accordance with previously published systematic reviews assessing prosthetic rehabilitation in the posterior part of the maxilla with short implants [11, 13–15]. However, the conclusions are often based on small patient populations, splinted prosthetic solutions or implant-supported prosthetic restorations involving both the mandible and maxilla as well as implants with a length of ≥ 8 mm [16, 17]. Moreover, in a recently published systematic review and meta-analysis, it was concluded that placement of short implants (< 8 mm) involves a greater risk of implant failure [13], which is in accordance with the conclusions of other reviews [18, 19].

From a clinical and patient perspective, it would be an advantage if the partially edentulous posterior part of the maxilla could be prosthetically rehabilitated with the use of short implants instead of standard length implants in conjunction with MSFA. A simplification of the surgical procedure will lead to improved acceptance from patients, diminishing the risk of donor site morbidity, and reduction of the financial costs [20]. However, single-crown restoration in the posterior part of maxilla with short implants (≤ 6 mm) compared with standard length implants in conjunction with MSFA have solely been assessed in few randomized, controlled clinical trials [21–25]. In the majority of the existing studies, short implants were splinted, and limited information is available on short implants supporting single-crown restorations in the posterior part of the maxilla. To provide more scientific data, this study was conducted. Therefore, the objective of the present randomized, controlled clinical trial was to test the H0-hypothesis of no difference in the clinical and radiologic treatment outcome of single-crown restorations supported by short implants (6 mm) compared with standard length implants (13 mm) in conjunction with MSFA after 1 year of functional implant loading.

## Materials and method

### Study population

**Fig. 1:**
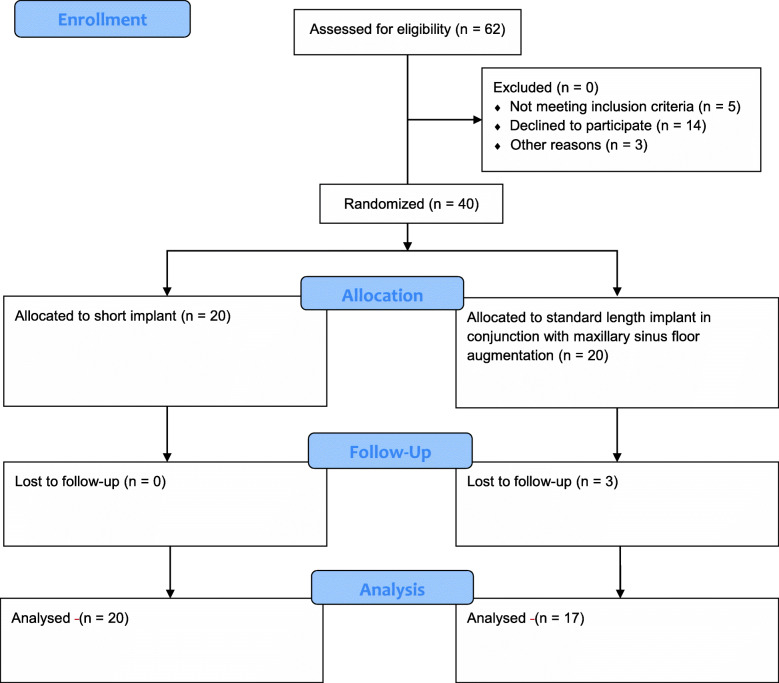
CONSORT flow diagram

The study was conducted at the Department of Oral and Maxillofacial Surgery, Aalborg University Hospital, Denmark. The study protocol was reviewed and approved by The North Denmark Region Committee on Health Research Ethics (Approval No.: 20160047). All potential participants received verbal and written information about the study by the principal investigator (HBN). An informed consent was signed by all participants before enrollment. CONSORT statement guidelines were followed (Fig. 1).

### Inclusion criteria

- Systemically healthy patients of ≥ 20 years of age
- Need of one implant in the posterior part of the maxilla
- Sufficient buccolingual bone width (≥ 8 mm)
- Missing posterior tooth in the maxilla for at least 4 months
- Residual alveolar bone height of a least 5.5 mm and less than 8 mm
- Sufficient mesial-distal dimension (7–9 mm)
- Presence of 7–10 mm of occlusal–gingival space to the opposing occluding dentition
- Presence of occluding mandibular teeth
- Able to understand and sign an informed consent form

### Exclusion criteria

- General contraindications to implant surgery
- Poor oral hygiene and motivation
- Progressive periodontitis
- Acute infection in the area intended for implant placement
- Parafunctional habits (Bruxism and/or clenching)
- Psychiatric problems or unrealistic expectations
- Pregnancy
- Heavy tobacco use (> 10 cigarettes per day)
- Substance abuse

Forty patients with partial edentulism in the posterior part of the maxilla were randomly allocated to implant treatment involving a single-crown restoration supported by a short implant (6 mm) (Astra Tech Implant System Osseospeed EV 4.2; Dentsply Sirona Implants, Mölndal, Sweden) or a standard length implant (13 mm) (Astra Tech Implant System Osseospeed EV 4.2; Dentsply Sirona Implants, Mölndal, Sweden) in conjunction with MSFA using 50% particulated autogenous mandibular bone graft from the ascending mandibular ramus mixed with 50% Bio-Oss (Geistlich Pharma AG, Wolhusen, Schwizerland) with a particle size 1–2 mm.

**Fig. 2:**
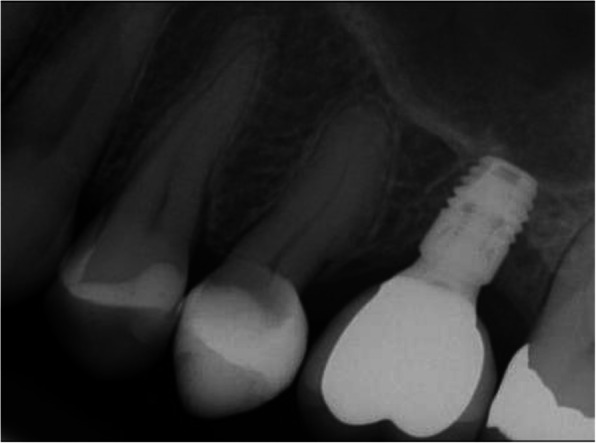
Periapical radiograph of a short implant at baseline

**Fig. 3:**
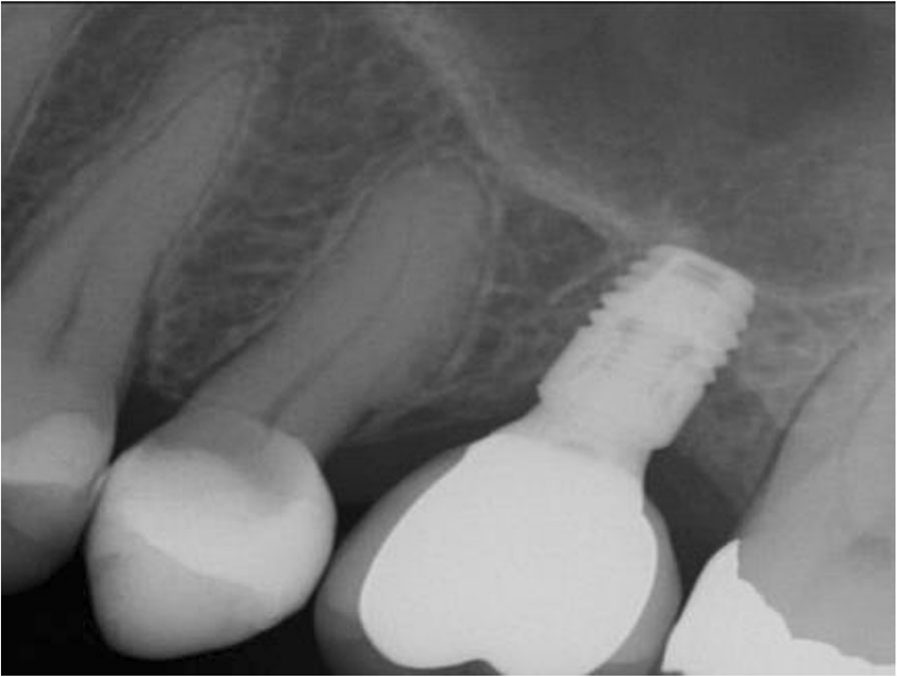
Periapical radiograph of a short implant one-year after functional loading

**Fig. 4:**
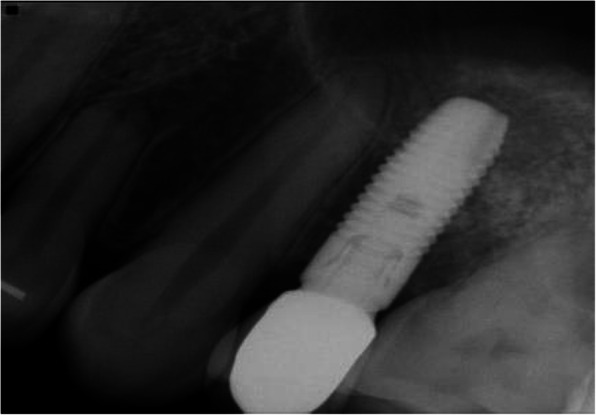
Periapical radiograph of a standard length implant in conjunction with MSFA at baseline

**Fig. 5:**
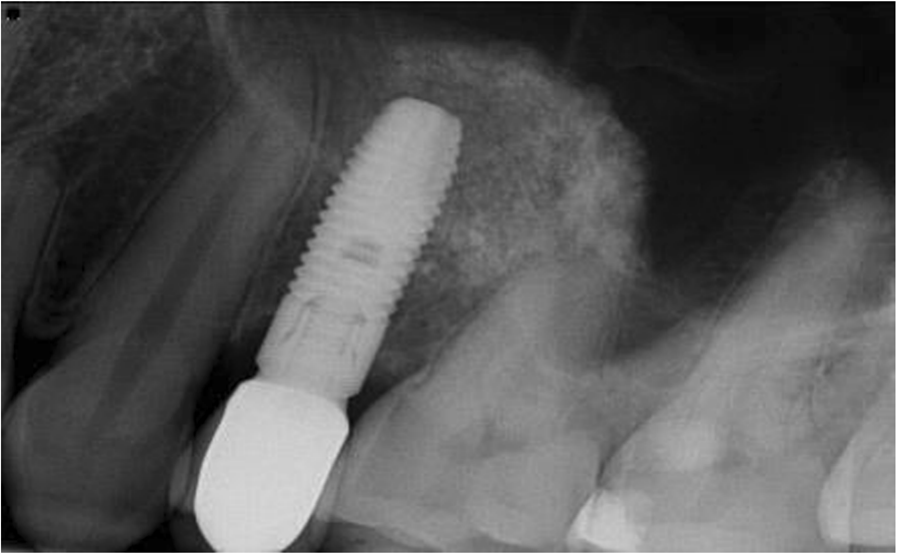
Periapical radiograph of a standard length implant in conjunction with MSFA one-year after functional loading

Preoperative clinical and radiographic examination included recordings of plaque index, bleeding on probing (BOP), and probing pocket depth (PPD) at the implant site and the neighboring tooth site. Periapical and panoramic radiographs were obtained for assessment of the marginal bone level of the neighboring teeth (Figs. 2, 3, 4, and 5) and measurements of the residual alveolar ridge height.

### Randomization

An independent block randomization schedule was generated in blocks of four (divisible by the number of study group) and designed to ensure a balanced distribution of treatments. The randomized treatment code was available in closed identical non-transparent sealed envelopes, and the patients were randomly assigned to treatment involving a single-crown restoration supported by a short implant or a standard length implant in conjunction with MSFA by pulling an envelope 1 week before surgery. Afterwards, the patient was informed about the surgical procedure to be applied. Blinding was not applicable due to the trial design.

### Description of surgical procedures

All treatment procedures were performed by the principal investigator (HBN).

One hour before implant placement, each patient was given prophylactic antibiotics involving Phenoxymethylpenicillin 2 MIE (Vepicombin Novum, Takeda Pharma A/S, Denmark) and Metronidazole 500 mg (Actavis A/S, Denmark). In case of penicillin allergy, Roxithromycin 300 mg (Surlid, Sanofi, Denmark) was used. Pain control involved Ibuprofen 400 mg (Burana, Teva, Denmark) and Paracetamol 1000 mg (Pamol, Takeda Pharma A/S, Denmark). Subsequently, each patient was asked to rinse with 0.2% Chlorhexidine solution (50 mg/l, GK Pharma, Denmark) for 1 min immediately before surgery.

After implant placement, all patients received antibiotics involving Phenoxymethylpenicillin 1 MIE, 1 tablet 3 times daily for 7 days and Metronidazole 500 mg, 1 tablet 3 times daily for 7 days. In case of penicillin allergy, Roxithromycin 300 mg, 1 tablet per day for 3 days was used. Postoperative pain control involved Ibuprofen 400 mg, 1 tablet 3 times daily and Paracetamol 500 mg, 2 tablets 4 times per day for 3–7 days. Detailed instructions on oral hygiene were provided, including Chlorhexidine mouthwashes 0.2% twice daily for 7 days. Finally, a soft diet was recommended for 2 weeks.

#### Short implants

A short implant was inserted under local anesthesia using Lidocaine (2%) with 1:200,000 adrenaline (Xylocaine, Amgros I/S, Denmark). The maxillary alveolar ridge was exposed via an incision at the top of the alveolar process combined with a marginal incision around the neighboring teeth and vertical releasing incision. Mucoperiosteum was reflected along the residual alveolar ridge, and the implant position was marked on the alveolar crest with a small round bur. An implant bed was successively prepared by standard implant protocol at 1200 rpm with saline irrigation according to the manufacturer’s recommendations. The integrity of the Schneiderian membrane was tested using Valsalva maneuver before a 6-mm implant (Astra Tech Implant System Osseospeed EV 4.2; Dentsply Sirona Implants, Mölndal, Sweden) was inserted with a cover screw. Periosteum and mucosa were sutured with Vicryl 4-0 (Ethicon FS-2, Ethicon, St-Stevens-Woluwe, Belgium). Sutures were removed 7–10 days after surgery. No provisional restoration was allowed during the healing phase.

#### Standard-length implants in conjunction with maxillary sinus floor augmentation

A standard-length implant in conjunction with MSFA was inserted under local anesthesia using Lidocaine (2%) with 1:200,000 adrenaline (Xylocaine, Amgros I/S, Denmark) and optional oral sedation, 5–10 mg Diazepam (Apozepam, Teva, Denmark), or general anesthesia with nasotracheal intubation. The region was exposed by a marginal incision from the second molar to the first premolar with a vertical releasing incision. Mucoperiosteum was reflected exposing the lateral wall of the maxillary sinus. A 1 × 1-cm window to the maxillary sinus was created with metal and diamond burrs maintaining an intact Schneiderian membrane. The Schneiderian membrane was carefully elevated from the maxillary sinus floor as well as the lateral sinus wall and displaced dorsocranially with blunt dissector. The implant bed was successively prepared following the manufacturer’s recommendations at 1200 rpm. A 13-mm implant (Astra Tech Implant System Osseospeed EV 4.2; Dentsply Sirona Implants, Mölndal, Sweden) was inserted with a cover screw.

The lateral surface of the mandibular ramus was exposed through an incision similar to the surgical approach for a sagittal mandibular ramus split osteotomy. Mucoperiosteum was elevated over the ascending mandibular ramus. A ramus clamp was secured over the coronoid process, before a 3 × 2 × 0.5-cm predominantly cortical bone graft was harvested from the outer cortex of the mandibular ramus using a horizontal osteotomy and two oblique cuts with fissure bur. The inferior osteotomy was performed incompletely through the cortex with a large round bur, before the osteotomies were completed with a bone chisel. The cortical plate was removed, and the wound was irrigated with saline and closed with Vicryl 4-0 (Ethicon FS-2, Ethicon, St-Stevens-Woluwe, Belgium). The autogenous bone graft was milled using a bone-mill (Roswitha Quétin Dentalprodukte, Germany) with 3-mm perforations to obtain bone graft particles with a size of 0.5–2 mm^3^. Specially prepared stainless steel cups of 1 cm^3^ were used to estimate a standardized equal distribution (50%) of particulated autogenous bone graft and Bio-Oss (Geistlich Pharma AG, Wolhusen, Schwizerland) with a particle size of 1–2 mm. The assistant dentist mixed the ratio of autogenous bone graft particles and Bio-Oss (Geistlich Pharma AG, Wolhusen, Schwizerland) (particle size 1–2 mm). The graft material was soaked in autogenous blood collected during the autogenous bone graft harvesting before the created cavity in the maxillary sinus around the implant was loosely packed with the graft material. The created window to the maxillary sinus was covered by a resorbable collagen barrier membrane (25 × 25 mm, Bio-Gide, Geistlich Pharma AG, Wolhusen, Schwizerland). Periosteum and mucosa were sutured with Vicryl 4-0 (Ethicon FS-2, Ethicon, St-Stevens-Woluwe, Belgium). Sutures were removed 7–10 days after surgery. No provisional restoration was allowed during the healing phase.

### Healing abutment connection

Healing abutment connection was performed under local anesthesia using Lidocaine (2%) with 1:200,000 adrenaline (Xylocaine, Amgros I/S, Denmark) 6 months after implant placement. The inserted implants were exposed by an incision slight palatinal of the alveolar crest. Mucoperiosteum was reflected, and the cover screw was removed. A prefabricated healing abutment (Dentsply Sirona Implants, Mölndal, Sweden) was placed after meticulous saline irrigation. The implant was tested manually for mobility and osseointegration by percussion. Finally, the mucosa was adapted and sutured with Vicryl 4-0 (Ethicon FS-2, Ethicon, St-Stevens-Woluwe, Belgium). Sutures were removed after 7–10 days and prosthetic restoration was initiated 3 weeks after healing abutment connection.

### Single-crown restoration

Prosthetic rehabilitation included individualized abutment (Atlantis, Dentsply Sirona Implants, Mölndal, Sweden) and a screw-retained single-crown restoration. Occlusal surfaces, protrusion, and laterotrusion were adjusted in slight contact with the opposite dentition. Patients were enrolled in an oral hygiene maintenance program with recall visits every 6 months. An accurate control of occlusion was performed involving evaluation of protrusion and laterotrusion. Moreover, maintenance care was provided. All prosthetic restorations and maintenance were performed in private practice by Dr. Connie Blauenfeldt, Aalborg Tandplejeteam, Aalborg, Denmark.

### Clinical outcome measures

Clinical examination was performed at baseline, after placement of the definitive crown, and 1 year after functional implant loading. Survival of suprastructure and implant, PIMBL, plaque index, (BOP), (PPD), biological, and mechanical complications were recorded at each visit.

#### Survival of suprastructure and implant

Loss of suprastructure was defined as a total loss due to mechanical and/or biological complications. Chipping of ceramics and loosening of the suprastructure were defined as mechanical complications and not categorized as loss of suprastructure.

Implant failure and loss of implants was defined as mobility of previously clinically stable or osseointegrated implants as well as removal of non-mobile implants due to progressive PIMBL and infection. Fracture of the implant or progressive PIMBL due to mechanical overload was also classified as implant loss. Implant loss was categorized as “early” (before connection of prosthetic abutment) or “late” (after connection of prosthetic abutment) failure.

#### Peri-implant marginal bone loss

PIMBL was estimated by linear measurements on digital periapical radiographs obtained at implant placement, baseline, and 1 year after functional implant loading. The distance from the implant-abutment connection to the PIMBL was measured mesially and distally parallel with the long-axis of the implant (ImageJ®, National Institute of Mental Health, Bethesda, MD, USA). Reference points for the linear measurements were the coronal margin of the implant shoulder and the most coronal point of bone-to-bone contact [26]. Magnification, brightness, contrast, and gamma adjustment was used for image enhancement.

### Statistical analyses and sample size

Data management and analysis was performed using STATA (StataCorp. 2019. Stata Statistical Software: Release 16. College Station, TX: StataCorp LLC.). Level of significance was 0.05. Sample size was determined using a power calculation based on differences in PIMBL changes performed in a previously published study involving replacement of a single tooth with 2 different protocols of implant treatment [27]. The calculation was based on the observed changes in PIMBL from insertion of the implant to abutment connection (A change of 0.65 mm and a standard deviation of 0.65), 17 patients in each group reached a power of 97% at the 5% level. With 15% to cover drop-outs, each treatment group included 20 patients. Patient demographics were reported as *n* when categorical and as mean, minimum, and maximum, otherwise. *p* values were found by Fischer’s exact test when categorical, and by Kruskal–Wallis test when continuous. Differences between values at different times were reported as mean and standard deviation and were compared using *t* test.

## Results

Forty patients (17 men and 23 women, mean age 52 years) were considered eligible and consecutively enrolled. Of the 40 patients enrolled, 37 completed the study. Demographic data (gender, age, residual alveolar bone height, smoking), preoperative periodontal health status, presence of a posterior tooth, crown-to-implant ratio, and distribution of implants according to their location are outlined in Table 1. There were no statistically significant differences in patient demographics, preoperative periodontal health status or presence of a posterior tooth. The difference in crown-to-implant ratio between the two treatment modalities was statistically significant (*p* ≤ 0.0495).

**Table 1:** Patient demographics

Parameter		Groups		***p*** value
	Patients	Short implants	Standard-length implants and maxillary sinus floor augmentation	
**Gender,** ***n***	37	20	17	0.74
Males	15	9	6	
Females	22	11	17	
**Age,** ***n***				1.00
20–35 years	5	3	2	
36–50 years	15	8	7	
51–65 years	14	7	7	
66–81 years	3	2	1	
**Smoking habits,** ***n***				1.00
Non-smokers	36	19	17	
Smokers	1	1	0	
**Mean residual bone height (mm, range)**	6.3 mm **(**5.5–7.0)	6.3 mm (5.5–7.0)	6.3 mm (5.5–7.0)	0.91
**Implant position,** ***n***				0.63
First premolar	6	3	3	
Second premolar	15	9	6	
First molar	22	10	12	
Second molar	2	2	0	
**History of periodontal disease,** ***n***				1.00
No history	32	17	15	
History	5	3	2	
**Distribution of implants,** ***n***				1.00
Single implant	30	16	14	
Multiple	7	4	3	
**Presence of posterior tooth/teeth**				0.61
None	4	3	1	
Presence	33	17	16	
**Crown-to-implant ratio (mm, range)**				
First premolar	1.39 (0.90–1.92)	1.87 (1.83–1.92)	0.90 (0.90–0.92)	0.0495
Second premolar	1.48 (0.86–2.00)	1.88 (1.70–2.00)	0.88 (0.86–0.88)	0.00
First molar	1.29 (0.83–1.91)	1.81 (1.70–1.91)	0.86 (0.83–0.90)	0.00
Second molar	1.80 (1.77–1.83)	1.81 (1.70–1.91)	- (.-.)	

### Survival of suprastructure and implants

The survival of suprastructure and implants was 100% for short implants and standard-length implants in conjunction with MSFA after 1 year of functional implant loading.

### Peri-implant marginal bone loss

PIMBL with the two treatment modalities after implant placement, at baseline, and after 1 year after functional implant loading are outlined in Table 2. There were no statistically significant differences in PIMBL with the two treatment modalities at any time point (*p* > 0.05).

**Table 2:** Peri-implant marginal bone loss over a 1-year period

PIMBL	Short implant Mean (SD)	Standard length implant and maxillary sinus floor augmentation Mean (SD)	p* value
**∆** IP–baseline	0.32 (0.15)	0.25 (0.12)	0.16
**∆** IP–1 year	0.60 (0.17)	0.51 (0.14)	0.09
**∆** Baseline–1 year	0.28 (0.17)	0.26 (0.14)	0.64

*IP* implant placement, *PIMBL* peri-implant marginal bone loss, *SD* standard deviation

*p value for same expected change from baseline to follow-up. Analysis by *t* test

### Clinical parameters of gingival inflammation

Plaque index, BOP, and PPD related to the two treatment modalities after implant placement, at baseline, and after 1 year after functional implant loading are outlined in Table 3. There were no statistically significant differences in the clinical parameters of gingival inflammation with the two treatment modalities at any time point (*p* > 0.05).

**Table 3:** Clinical parameters of gingival inflammation over a 1-year period

	Short implant Mean (SD)			Standard length implant and maxillary sinus floor augmentation Mean (SD)			***p value***
	Implant placement	Baseline	1 year	Implant placement	Baseline	1 year	
PI	1.62 (0.6)	1.46 (0.4)	1.32 (0.4)	1.68 (0.5)	1.38 (0.5)	1.36 (0.3)	0.52
PPD	2.80 (0.8)	2.6 (0.6)	2.4 (0.5)	2.9 (0.5)	2.7 (0.4)	2.5 (0.6)	0.41
BOP (%)	38	27	24	36	28	22	0.64

*BOP* bleeding on probing, *PI* plaque index, *PPD* probing pocket depth

### Biological and mechanical complications

#### Short implant

No biological complications were noted. Mechanical complications occurred in two patients including chipping of ceramics and abutment screw loosening (Table 4).

**Table 4:** Biological and mechanical complications

	Patients,	Short implant, ***n***	Standard length implant and maxillary sinus floor augmentation, ***n***	p* value
**Biological complications**	11	0	11	0.00
Intraoperative bleeding	1	0	1	0.46
Perforation of the Schneiderian membrane	3	0	3	0.09
Pain and swelling	4	0	4	0.04
Extensive cicatricial soft tissue	1	0	1	0.46
Infection	1	0	1	0.46
Permanent neurosensory disturbance	1	0	1	0.46
**Mechanical complications**	8	2	6	0.13
Abutment screw loosening	4	1	3	0.34
Loss of abutment	1	0	1	0.49
Loosening of suprastructure	1	0	1	0.49
Chipping of ceramics	2	1	1	1.00

*Fischer’s exact test

#### Standard-length implants

Eleven biological complications were noted (Table 4). Biological complications included intraoperative complications (perforation of the Schneiderian membrane and bleeding), immediate postoperative complications (pain, swelling, and infection), and late postoperative complications (extensive cicatricial tissue and neurosensory disturbances). Intraoperative perforation of the Schneiderian membrane occurred in three patients. The size was less than 2 mm in all cases, and the perforation was covered with a resorbable collagen membrane (Bio-Gide, Geistlich Pharma AG, Wolhusen, Schwizerland). Intraoperative bleeding occurred in one patient. Pain and swelling with a duration of more than 1 week occurred in four patients. A late postoperative infection occurred in one patient, which were treated successfully with additional antibiotics, including Phenoxymethylpenicillin 1 MIE (Vepicombin Novum, Takeda Pharma A/S, Denmark), 1 tablet 3 times per day and Metronidazole 500 mg (Actavis A/S, Denmark), 1 tablet 3 times per day for 7 days. Extensive cicatricial tissue after autogenous bone harvesting from the ascending mandibular ramus was seen in one patient and persistent neurosensory disturbance of the inferior alveolar nerve was observed in one patient after 1 year.

Mechanical complications occurred in six patients (Table 4). Healing abutment screw loosening were seen in three patients and loss of healing abutment occurred in one patient. Loosening of the suprastructure was seen in one patient, and chipping of ceramics occurred in one patient.

Standard length implants in conjunction with MSFA revealed a statistically significant higher incidence of biologic complications compared with short implants (*p* = 0.00). There were no statistically significant differences in mechanical complications between the two treatment modalities (*p* = 0.13).

## Discussion

Single-crown restorations supported by short implants (6 mm) compared with standard-length implants (13 mm) in conjunction with MSFA were assessed after 1 year of functional implant loading in the present study. No statistically significant difference in survival of suprastructure or implants, PIMBL, and mechanical complications was revealed. However, standard-length implants in conjunction with MSFA disclosed a statistically significant higher incidence of biological complications including perforation of the Schneiderian membrane, bleeding, swelling, and pain. Moreover, a permanent neurosensory disturbance of the inferior alveolar nerve was observed in one patient after harvesting of autogenous bone graft from the ascending mandibular ramus.

Previously published systematic reviews and meta-analyses have demonstrated no statistically significant differences in the survival rate of suprastructure and implants after prosthetic rehabilitation of the posterior part of the maxilla with single-crown restorations supported by short implants compared with standard-length implants in conjunction with MSFA [11, 12, 14]. However, standard-length implants in conjunction with MSFA were characterized by a non-significant higher PIMBL and more biological complications [12, 13, 24]. These results are in accordance with the present study.

A newly published long-term randomized, controlled clinical trial assessing single-crown restorations in the posterior part of the maxilla with short implant (6 mm) compared with standard-length implants (11–15 mm) in conjunction with MSFA demonstrated an implant survival rate of 98.5% with short implants and 100% with standard-length implants after 5 years of functional implant loading [25]. Limited PIMBL was revealed with both treatment modalities without any statistically significant differences, but standard-length implants in conjunction with MSFA were associated with a higher incidence of biological complications compared with short implants [25]. These long-term results are in accordance with the results of the present short-term study.

Biological complications including perforation of the Schneiderian membrane, sinusitis, infection, loss of grafting material, pain, swelling, and intra- and postoperative bleeding are frequently reported after MSFA as documented in several systematic reviews and long-term studies [2, 4–6]. Moreover, harvesting of autogenous bone graft from the mandible in conjunction with MSFA is associated with risk of donor site morbidity involving neurosensory disturbance of the inferior alveolar nerve [5, 10, 28]. Previous studies have estimated temporary neurosensory disturbances of the alveolar inferior nerve due to harvesting of bone from the ascending mandibular ramus up to 19.6% [29] and permanent neurosensory disturbances of the alveolar inferior nerve up to 2.3% [30]. Consequently, placement of standard-length implants in conjunction with MSFA is associated with an increased risk of biological complications as well as risk of donor site morbidity related to the harvesting procedure [31]. Potential risks of biological complications associated to MSFA are an important part of the initial treatment planning among other factors such as financial aspects, operator experience and treatment time. In general, two treatment modalities rendering a similar treatment outcome, the most cost-effective treatment modality appears more favorable.

Mechanical complications including screw loosening, loss of retention, and chipping of ceramics are frequently reported after prosthetic rehabilitation with implant-supported single crowns [32, 33]. Moreover, more than half of the patients with mechanical complications experience more than one mechanical complication [34]. A long-term randomized, controlled clinical trial demonstrated more mechanical complications after single-crown restoration in the posterior maxilla with short implant (6 mm) compared with standard-length implants (11–15 mm) in conjunction with MSFA after 5 years of functional implant loading [25]. These results are in contrast with the present study demonstrating no statistically significant differences in mechanical complications between the two treatment modalities.

Bruxism and/or occlusal overloading may cause increased risk of mechanical complications including failure of suprastructure and implant [35–38]. Moreover, occlusal overloading in the presence of inflammation significantly increased the risk of PIMBL [39]. Thus, placement of short implants necessitates meticulous examination of the periodontal health status and establishment of a balanced functional occlusion combined with a regular oral hygiene maintenance program [35, 40, 41]. In the present study, all included patients were examined by an experienced dentist before implant placement ensuring optimal periodontal health status and balanced functional occlusion. After prosthetic rehabilitation, the patients were seen annually for maintenance of the periodontal health and evaluation of the occlusion.

Placement of short implants increases the crown-to-implant ratio compared with placement of standard length implants. Previous studies have indicated that a higher crown-to-implant ratio may be detrimental to the long-term implant survival and aggravate PIMBL due to an unfavorable occlusal force and stress distribution to the peri-implant marginal bone [42–44]. However, it has been concluded in several long-term studies and systematic reviews that an increased crown-to-implant ratio does not seem to be directly related with an increased risk of implant loss, PIMBL, or mechanical complications [45–50]. Moreover, it was concluded in a newly published systematic review assessing short implants in the posterior part of maxilla that a higher crown-to-implant ratio was not associated with increased risk of implant loss and PIMBL [51]. In the presented study, the difference between the two treatment modalities according to crown–implant ratio was statistically significant (*p* ≤ 0.049) disclosing no statistically significant differences in implant survival, PIMBL, and mechanical complications were revealed. The influence of crown-to-implant ratio on implant survival, PIMBL, and mechanical complications is therefore still controversial, and the conclusions of previous studies are often based on splinted prosthetic solutions in the maxilla and mandible with a balanced functional occlusion [4, 47, 51]. Therefore, further long-term studies assessing a correlation between increased crown-to-implant ratio on implant survival, PIMBL, and mechanical complications after single-crown restorations supported by short implants in the posterior maxilla are needed [52].

The design of the present study is characterized by various limitations including a small sample population, short-term observation period, and no blinding of participants or treatment providers. Therefore, further long-term randomized, controlled clinical trials with larger patient samples are needed before one treatment modality might be considered superior to another.

## Conclusions

Within the limitations of the present short-term study, it can be concluded that single-crown restorations supported by short implants (6 mm) seems to be a comparable treatment modality to standard-length implants (13 mm) in conjunction with MSFA for prosthetic rehabilitation of the posterior part of the maxilla after 1 year of functional implant loading. High survival rates of suprastructure and implants, limited PIMBL, and no statistically significant differences in mechanical complications were revealed with both treatment modalities. However, placement of standard-length implants in conjunction with MSFA were associated with a higher incidence of biological complications. Therefore, further long-term randomized, controlled clinical trials including assessment of donor site morbidity, patient-reported outcome measures, economic perspective, and length of treatment time are needed before definite conclusions can be provided about the two treatment modalities.

## Data Availability

The authors of this work are available to support the data.

## Abbreviations

BOP: Bleeding on probing
MSF: Maxillary sinus floor augmentation
PIMBL: Peri-implant marginal bone loss
PPD: Probing pocket depth
